# Effect of *KLF15*-Mediated Circadian Rhythm on Myocardial Infarction: A Narrative Review

**DOI:** 10.3390/ijms26104831

**Published:** 2025-05-18

**Authors:** Junxin Zhao, Zhuoyang Chen, Jingyi Yang, Lincheng Duan, Hong Yang, Dingjun Cai, Zhengyu Zhao

**Affiliations:** Acupuncture and Tuina School, Chengdu University of Traditional Chinese Medicine, Chengdu 610075, China; xiaoyuzhao631@gmail.com (J.Z.); chenzhuoyang2025@163.com (Z.C.); yangjingyi@stu.cdutcm.edu.cn (J.Y.); lincheng@stu.cdutcm.edu.cn (L.D.); yanghong95ob@163.com (H.Y.)

**Keywords:** circadian rhythm, *KLF15*, myocardial infarction, biological clock, cardiovascular disease

## Abstract

Normal circadian rhythms are essential for organisms to adapt to diurnal changes and maintain an optimal state of physiological function. Disturbances in circadian rhythms such as shift work and working at night increase the risk of cardiovascular disease. Myocardial infarction exhibits a marked circadian rhythm, usually peaking in the early morning. Krüppel-like factor 15 (KLF15), a transcription factor with a circadian rhythm, plays an important role in cardiac physiopathology. It has a protective effect against myocardial injury after myocardial infarction by regulating energy metabolism and inflammatory factors, among other pathways. Currently, the association between circadian rhythm, *KLF15*, and myocardial infarction is unclear, thus this paper reviews how circadian rhythm influences the role of *KLF15* in myocardial infarction, aiming to reveal the association between circadian rhythm, *KLF15*, and myocardial infarction, and to explore the underlying mechanisms, to provide new theoretical insights and therapeutic strategies for the clinical treatment of myocardial infarction.

## 1. Introduction

Myocardial infarction is a critical disease resulting from acute occlusion of coronary arteries leading to myocardial ischemia, hypoxia, and necrosis, which in turn severely affects cardiac function. As one of the leading causes of death and disability worldwide, its morbidity and mortality rates continue to rise, posing a major threat to public health [1,2]. The high cost of treatment not only aggravates the economic burden of patients’ families, but also creates a heavy social medical burden, which makes the study of myocardial infarction particularly urgent. Clinical symptoms of myocardial infarction include chest pain from the left arm to the neck, shortness of breath, sweating, nausea and vomiting, abnormal heartbeat, anxiety and depression, and weakness [3,4]. A definitive diagnosis of myocardial infarction is made when a patient presents with an increase or decrease in cardiac biomarkers (e.g., troponin) with at least one value higher than 99% of the reference value, accompanied by at least one symptom of myocardial ischemia.

The occurrence of myocardial infarction is closely related to a number of risk factors, the traditional risk factors include advanced age, hypertension, diabetes, and obesity. However, it is worth noting that the incidence of myocardial infarction in young people under the age of 45 has been on the rise in recent years. Unlike older patients whose morbidity is mostly due to underlying diseases, the main risk factors for younger patients are more inclined to poor lifestyle (e.g., lack of exercise, prolonged staying up late) and inappropriate stress coping styles (including smoking, alcohol abuse, and drug abuse). In particular, staying up late is one of the most important factors in the development of myocardial infarction in young people [5]. This is associated with the involvement of circadian rhythm-related genes in regulating the physiological activities of the heart [6,7], such as heart rate, blood pressure, and myocardial contractility which fluctuate throughout the day, peaking in the afternoon and troughing at night [8]. These changes are regulated by the autonomic nervous system in addition to the central biological clock, which, by influencing the vasculature and peripheral biological clocks in the myocardial cells, ensures that the heart’s rhythm matches that of the whole organism [9,10]. The cardiac biological clock consists of circadian oscillators, circadian input/output pathways, and circadian regulators. Rhythm oscillators are the core components that influence cyclic changes in gene expression and protein levels to regulate physiological processes within the organism [11]. The core oscillator, in turn, is composed of multiple transcriptional-translational feedback loops of genes and proteins [11] that are tightly coupled to systemic signals (e.g., hormones, autonomic inputs) and local factors (e.g., metabolic demands) that fine-tune cardiac physiology throughout the 24-h cycle. Circadian input pathways regulate and reset the biological clock by receiving signals from the outside world [12], the most important of which is light. Natural light is detected by cells in the retina’s specialized photoreceptors, and the signals are transmitted to the hypothalamus’s suprachiasmatic nucleus (SCN), which then transmits the signals to other areas of the brain and peripheral tissues including the heart [13]. In the cardiovascular system, the SCN transmits circadian signals to the heart through hormonal signaling (e.g., glucocorticoid or melatonin-mediated signaling) and the autonomic nervous system (sympathetic and parasympathetic nerves) [14,15,16], which ensures that the cardiac peripheral bioclocks’ rhythmic activity is synchronized with the external light-dark cycle, thus optimizing the cardiac electrophysiologic properties metabolic activity and contractile function to adapt to the demands of daily activity patterns [17]. A disturbance in this cascade of regulation (e.g., suprachiasmatic nucleus with the peripheral biological clock) can lead to cardiac rhythmic dysregulation and increase the risk of cardiovascular diseases such as arrhythmias and heart failure.

*KLF15*, an important rhythm gene in the heart [18], whose expression is regulated by core clock genes such as *Bmal1*, exhibits a clear pattern of circadian oscillations [19]. It has been found [19] that *KLF15* regulates biphasic transcriptional oscillations of genes in the heart: one phase corresponds to the active phase in rodents, dominated by maximization of energy supply, and the other phase corresponds to the resting phase, dominated by cardiac remodeling and repair. However, in the case of normal core clock gene rhythms, this phase assignment becomes disordered when the rhythm of *KLF15* is disrupted, this shows that *KLF15* is the node between the heart and biological clock rhythms. And it has been found [20,21] that the onset of myocardial infarction is characterized by a significant circadian rhythm, with its incidence peaking in the early morning hours. Is there a link between the onset of myocardial infarction and *KLF15* and circadian rhythm? Therefore, the purpose of this paper is to explore the relationship between the three and their potential mechanisms, with a view to providing new perspectives for the prevention and treatment of myocardial infarction.

Objectives: This review systematically investigated (1) the circadian rhythmicity of *KLF15*, (2) the role of *KLF15* in the physiology and pathology of myocardial infarction, and (3) the therapeutic potential of modulating *KLF15*.

## 2. Methodology

### 2.1. Literature Search Strategy

The literature search for this review encompassed comprehensive coverage of international authoritative databases, including PubMed, Web of Science, and Embase, as well as Chinese databases, such as China National Knowledge Infrastructure (CNKI), Wanfang, and China Science and Technology Journal Database (VIP), to ensure a thorough retrieval of both English and Chinese publications. The search strategy was developed using the core terms “*KLF15*”, “circadian rhythm”, and “myocardial infarction” derived from the Medical Subject Headings (MeSHs) thesaurus, with no time restrictions applied to fully integrate historical foundations and recent advancements in the field. The literature screening prioritized thematic relevance, emphasizing the inclusion of highly cited original studies, reviews, and methodological papers. Manual searches of high-impact journals within the field were additionally performed. Although this narrative review did not adhere to the standardized search protocols of systematic reviews, cross-verification across multiple databases and iterative keyword optimization ensured the representativeness of the selected literature in elucidating the mechanistic links between *KLF15*, circadian rhythms, and myocardial infarction. This approach established a robust evidence base to support subsequent theoretical explorations.

### 2.2. Key References Table

The key references for this review are listed in Table 1.

## 3. Effect of *KLF15*-Mediated Circadian Rhythm on Myocardial Infarction

### 3.1. Myocardial Infarction Is Characterized by a Significant Circadian Rhythm

Epidemiological investigations have shown that the occurrence of sudden cardiac death, myocardial infarction, and stroke have circadian rhythms, in which myocardial infarction is mostly concentrated in the early morning from 6–9 a.m., and some scholars believe that neural awakening and endogenous risk factors in the early morning hours are one of the reasons for the high prevalence of myocardial infarction [22]; moreover, elevated blood pressure, heart rate, coagulation activity, and vascular tone underlie the high incidence of this disease, and the blood pressure is often higher in the daytime than at night [48], and the overall trend is higher in the morning and lower in the evening, but this rhythm disappeared in mice knocked out of the *Bmal1* or *CLOCK* genes, suggesting that changes in blood pressure have a circadian rhythm; as a cardioprotective agent, natriuretic peptide also has a circadian rhythm, which makes it able to offset the cardiac damage produced with the circadian changes in blood pressure [23]. The vascular endothelium, which maintains blood flow to vital organs, is regulated by endothelin-1 [24] and oxidative stress [25] and both biomarkers also have significant circadian rhythms, with increases in both biomarkers in the morning increasing the risk of cardiovascular disease; coagulation activity is also enhanced in the morning, as evidenced by elevated levels of prothrombotic fibrinogen activator inhibitor-1 [26] and platelet surface activity GPIIb-IIIa (the last part of the platelet aggregation pathway) [27], which may increase the likelihood of thrombosis and the risk of myocardial infarction by the appearance of a morning peak on waking up in the morning [49].

### 3.2. Effect of Circadian Rhythm Disturbances on Myocardial Infarction

Long late nights and shift work have become the norm in today’s society, significantly increasing the risk of myocardial infarction in young and middle-aged people, and possibly even leading to sudden death [28]. This is due to the fact that circadian rhythm disruption contributes to elevated basal reactive oxygen species (ROS), elevated levels of thiobarbituric acid reactive substances (TBARS), and hydrogen peroxide in the body. At the same time, it leads to a decrease in antioxidant capacity, prompting the body’s oxidative stress to be more intense, and cardiomyocytes are more prone to ischemic stress and hypoxia-reoxygenation injury, increasing myocardial cell death [29,50], therefore, it is more likely to suffer from heart disease than the general population [51,52]. In an earlier experiment, TBARS and Glutathione peroxidase (GPx) were observed to oscillate throughout the day in the rat heart, with GPx concentrations decreasing and TBARS concentrations increasing in the evening [53]. This phenomenon also suggests that the mismatch between high ROS production and low antioxidant capacity may reduce the heart’s response to oxidative stress in a time-dependent manner, thereby impairing cardiac adaptation to oxidative stress and causing damage.

Circadian rhythm disturbances also have an impact on the prognostic outcome of patients with myocardial infarction. The prognosis of mice recovering from experimentally induced myocardial ischemia is disturbed by rapidly changing light/dark cycles. After myocardial infarction occurs, the body repairs the heart in order to remove dead cells and debris by triggering an inflammatory response that activates cardiomyocyte fibrosis to form a scar [54,55,56]. The circadian mechanism plays a key role in the process of recruiting immune cells [30]. When the biological clock gene is impaired, oxidative stress, mitochondrial dysfunction, and ultimately cardiomyocyte death occurs. However, when gene expression is restored, it improves cardiac function and cellular activity by upregulating cellular autophagy [31]. A similar phenomenon can also affect the recovery of hospitalized patients [32]. Data have also shown that in mice knocked out of the cardiac-specific *Bmal1* gene, the level of genes involved in oxidative stress, including GPx, superoxide dismutase, and oxygen transport proteins, is enhanced, suggesting that disruption of the genetic mechanisms of circadian rhythms triggers oxidative stress in the heart [33], and that when circadian rhythms are disrupted, it worsens the response to myocardial infarction [42,57].

### 3.3. KLF15 Gene: A Central Regulator of Cardiac Circadian Rhythm

*KLF15*, as the only recognized peripheral biological clock in the heart, is essential for the regulation of cardiac physiological functions. Darwin Jeyaraj et al. conducted experiments on mice with light-dark cycle rotation every 12 h and found that there are rhythmic oscillations of *KLF15* in peripheral tissues and organs, such as the heart, liver, and skeletal muscle, and that *KLF15* is able to regulate the rhythmic expression of about 75% of the genes occurring in the heart, which is needed to maintain normal physiological function of the heart [19,34]. Chip-seq found that *Bmal1* is rhythmically expressed at the promoter of *KLF15*, indicating that *KLF15* expression is regulated by the Endogenous Core Clock Mechanism (ECM) [35], suggesting that *KLF15* is characterized by endogenous circadian oscillations. In addition, *KLF15* is involved in regulating the expression of cardiac ion channels and endogenous circadian rhythm of the QT interval, thus maintaining the relationship between normal ventricular rhythm and circadian rhythm. Thus, when *KLF15* expression is abnormal, it leads to disruption or loss of rhythmic oscillations of most genes in the heart, which, in turn, affects the cardiac ion channel expression and the endogenous circadian rhythm of the QT interval, inducing the appearance of ventricular arrhythmia. It has also been noted that arrhythmias are associated with a higher incidence of myocardial infarction in the early morning period [18]. It has been found that when *KLF15* is knocked out or the rhythm expression is disturbed when the core clock genes are normally expressed, the orderly gene oscillations presented in the heart are disrupted, proving that *KLF15* is the hub between the biological clock rhythms and the heart. This shows that the external circadian rhythm can affect the rhythm of *KLF15* through the core clock gene, and the circadian rhythm of *KLF15* can affect the incidence of myocardial infarction, so there is a correlation between the three.

### 3.4. The Role of KLF15 in the Pathologic Process of Myocardial Infarction

#### 3.4.1. Modulation of the Inflammatory Response

It has been found that when myocardial infarction occurs, pro-inflammatory factors in cardiomyocytes are increased, such as IL-6, TNF-α, IL-1β, signaling that the NF-κB signaling pathway may be activated and involved in myocardial inflammatory response [36]. *KLF15* can inhibit NF-κB transcriptional activation and attenuate inflammatory response by inhibiting p300-mediated acetylation [58]. *KLF15* can also inhibit MAPK transcriptional activation by inhibiting P38 and ERK1/2 [37]. Furthermore, WWP1 can catalyze K48-linked polyubiquitination and degradation by targeting *KLF15*, triggering excessive cardiomyocyte inflammation after myocardial infarction. Twist2 acts as a transcription factor with anti-inflammatory and attenuating endoplasmic reticulum stress, ameliorating mitochondrial content and oxidative stress functions, *KLF15* can directly regulate the promoter of Twist2 and enhance transcription, thereby exerting its function to reduce myocardial injury [38]. In addition, *KLF15* can also alleviate post-ischemic reperfusion injury by regulating CTRP12 [39]. *KLF15* is also a direct target of *miRNA-223-3p* and *miRNA-137-3p*, both of which can reduce the expression of *KLF15* by targeting inhibition of *KLF15*, increase cardiomyocyte apoptosis and oxidative stress, and exacerbate ischemia-reperfusion injury [59,60]. In contrast, *KLF15* expression can be increased by inhibiting the above two RNAs, thus playing a therapeutic role. Thus, *KLF15* can regulate the inflammatory response after myocardial infarction through various pathways such as NF-κB, MAPK, Twist2, and CTRP12 [61].

#### 3.4.2. Inhibition of Apoptosis

Previous studies have confirmed that *KLF15* is involved in the regulation of apoptosis in a variety of diseases. For example, *KLF15* overexpression promotes the proliferation and metastasis of H/R-induced HTR8/SVneo cells by mediating the PI3K-AKTpathway, and inhibits oxidative stress and apoptosis [40]. In cellular experimental studies of a gastric cancer model, overexpression of *KLF15* promotes apoptosis and autophagy through PI3K/Akt/ mTOR promoted apoptosis and autophagy, and inhibited the proliferation and invasion of gastric cancer cells [62]. *KLF15* was able to attenuate lipopolysaccharide-induced apoptosis and inflammatory response in renal tubular epithelial cells by interacting with PPARδ [63].

*KLF15* is widely expressed in cardiomyocytes and fibroblasts of the heart [64]. A 2020 study found [34] that mice knocked down for *KLF15* had a larger infarcted area of the heart after modeling compared to normal mice, suggesting the importance of *KLF15* for cardioprotection. *MiRNA-223-3p* negatively regulated the expression of *KLF15*, leading to increased apoptosis in cardiomyocytes, whereas silencing miR-223-3p could upregulate *KLF15* expression to attenuate oxidative stress damage to H9c2 cells. At the same time, this process also prevents cardiomyocyte apoptosis and protects cardiomyocytes from hypoxic injury by decreasing the expression of Bax and C-caspase3 and increasing the expression of Bcl-2 [65]. Xialu’s study [66] confirms that the inhibition of *KLF15* ubiquitylation can effectively alleviate the apoptosis induced by the increase of WWP1 expression after myocardial infarction.

#### 3.4.3. Regulation of Energy Metabolism

Studies have shown that *KLF15* regulates nutrient fluxes to maintain energy homeostasis in fasting or exercising organisms under normal circadian rhythms [35,41,65,66]. *KLF15* is closely related to several key metabolic pathways [67,68], such as glucose metabolism, fatty acid metabolism, and amino acid metabolism, and is involved in the regulation of myocardial ischemia, cardiomyocyte hypertrophy, and fibrosis, and plays an important role in cardiovascular system development, ischemic adaptation, and cardiac remodeling [69].

##### Glucose Metabolism and Cardiac Energy Supply

*KLF15*, as an important transcription factor that activates gluconeogenesis-related genes, is able to maintain the body in a normal glycemic state under fasting. *KLF15* releases alanine and other amino acids into the circulation for hepatic uptake through the catabolism of skeletal muscle branched-chain amino acids (BCAAs) and up-regulates key enzymes of BCAA catabolism to inhibit lipogenesis and promote gluconeogenesis, thereby providing the liver with a substrate for gluconeogenesis [70]. Myocardial glucose uptake is mainly carried out under Glucose Transporter Type 4 GLUT4, which can promote myocardial glucose uptake when the body is in ischemia, exercise stimulation or insulin secretion is insufficient. It has been shown that *KLF15* can promote the entry of glucose into cardiomyocytes by regulating insulin secretion [42], thus providing energy for the physiological activities of the heart. Some studies have found that glucocorticoids can promote glucose metabolism in the heart by activating *KLF15*, and glucocorticoid (GC) is the pathway of circadian signaling from SCN to the heart, and its own circadian rhythm can mimic the effect of the external circadian rhythm on *KLF15*, which shows that circadian rhythm can affect the regulation of glucose metabolism by *KLF15* [43].

##### Homeostatic Maintenance of Lipid Metabolism

*KLF15* is a key regulator of cardiac lipid metabolism. From birth, *KLF15* accumulates in the heart and decreases in heart failure, a trend similar to the pattern of lipid oxidation. *KLF15* activates its direct transcriptional targets by interacting with p300, which in turn affects lipid flux and metabolic homeostasis [41]. In patients with heart failure, the heart’s ability to metabolize lipids is reduced, leading to excessive lipid deposition within the heart. *KLF15* negatively regulates cardiac lipid deposition and participates in the process of cardiac functional recovery after unloading [44]. Muscle-specific *KLF15* knockout mice were found to have significantly reduced lipid utilization and were more prone to obesity [66,71]. PPAR-δ and PPAR-γ, as members of the Peroxisome Proliferator-Activated Receptor (PPAR) family, can regulate adipocyte differentiation and lipid metabolism, and it has been demonstrated that *KLF15* directly affects the regulation of lipid metabolism in muscle tissues by PPAR-δ [72], and that *KLF15* can regulate the expression of PPAR-γ to inhibit adipogenesis [45,73].

A large part of the energy supply of the adult heart relies on fatty acid oxidation supply, especially in the fasting state, *KLF15* can affect fatty acid oxidative catabolism for energy support by regulating the oscillatory expression of cardiac metabolic genes [74,75]. In summary, *KLF15* can regulate lipid metabolism in the organism and cardiac tissues, and when *KLF15* is absent or dysrhythmic, the ordered rhythmic oscillations are abnormal, thus affecting the energy supply to the body and heart.

##### Circadian Rhythmicity of Branched-Chain Amino Acid Metabolism

Branched-chain amino acids (BCAAs) play an important role in muscle energy metabolism, and it has been found that *KLF15* is able to drive the activation of BCAA to accelerate proteolysis [76], whereas GC is able to prompt *KLF15* to exacerbate the activation of BCAA, further accelerating proteolysis, suggesting that, at the peak of the rhythmic expression of GC, it is able to increase the metabolic rate by increasing the expression of *KLF15*. It has also been shown that, when *KLF15* levels decline, BCAA key metabolic enzymes are also absent, and the decline or absence of both of the above, in a mouse model of heart failure, leads to a large accumulation of branched-chain keto acids (BCKAs), which in turn negatively affects mitochondrial function [46]. These indirectly demonstrate that circadian rhythmic expression of *KLF15* likewise affects the degree of activation of BCAA metabolism. In addition, a study in Chinese sea bass found that fasting led to a disturbance in the rhythmic expression of *KLF15* [47]. Whereas the circadian rhythmicity of BCAA metabolism exists in a *KLF15*-dependent manner [77]. *KLF15* can also act as a rhythmic oscillatory regulator of nitrogen flux and homeostasis, mammals need to remove toxic metabolic by-products by converting ammonia to urea, and it has been found that the expression of genes involved in the metabolism of amino acids and the urea cycle has a rhythmicity [78,79], whereas *KLF15* is able to regulate nitrogen homeostasis in mice with a circadian rhythm, and when *KLF15* is absent and the rhythm is disturbed, the total amino acid and urea levels in plasma are altered [35], and the concentration of BCAA is elevated, which proves that *KLF15* and its circadian rhythmicity are able to influence the metabolic changes of BCAA and nitrogen homeostasis in vivo.

In summary, under physiological conditions, *KLF15* can influence multiple energy metabolic pathways in the body and the heart through its own circadian rhythmic characteristics, to maintain energy demand during the active period, and, at the same time, during the resting period, to alleviate the inflammatory response and apoptosis, to repair daily damage to the heart. However, when *KLF15* is absent or the rhythm is disturbed, it will result in the heart not being able to repair the damage caused by inflammation and apoptosis in a timely manner, leading to both an increased risk of myocardial infarction and loss of restraint and repair of pathologic damage after myocardial infarction. In addition, it also affects the energy metabolism of the heart, leading to an inadequate supply of energy metabolism and exacerbating myocardial infarction damage [Table 2].

## 4. Discussion

**Figure 1:**
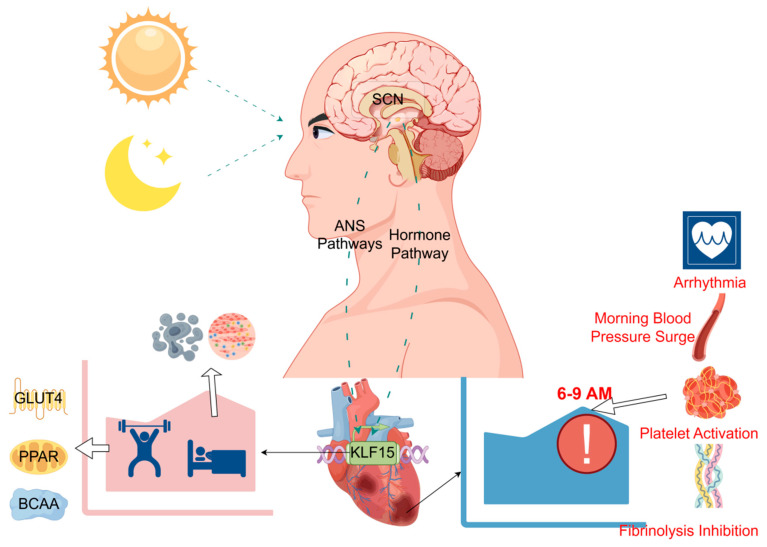
*KLF15*-mediated regulation of cardiac circadian rhythm and risk of myocardial infarction.

*KLF15* is downstream of the core clock gene *Bmal1* and is influenced by it, making its own expression characterized by a significant circadian rhythm. As a hub between the biological clock and cardiac genes, its own rhythmic expression can influence the normal circadian rhythm of most gene expression in the heart under physiological conditions. The unique biphasic clustering of gene functions it generates within the heart allows the heart to obtain an adequate supply of energy during physical activity and to repair cardiac damage at rest [19], ensuring the health of the heart. However, when the circadian rhythm is disturbed or *KLF15* is deficient, the ion channels of the heart become dysregulated, leading to the occurrence of arrhythmia and laying a hidden danger for the occurrence of myocardial infarction. In addition, the abnormality of the two will cause the normal energy metabolism to be disrupted, resulting in insufficient energy supply to the heart, unable to cope with the energy crisis after myocardial infarction, exacerbating the death of cardiac myocytes, resulting in more serious post-infarction damage. At the same time, the inflammatory response and oxidative stress, which were previously under the rhythmic control of *KLF15*, were also abnormally aggravated, causing more serious myocardial damage and accelerating the process of cardiac failure after myocardial infarction. In the course of myocardial infarction treatment, whether the circadian rhythm is normal or not, and whether the *KLF15* expression is increased or not, play an indispensable role in the prognosis of the patients. It has been found that patients with myocardial infarction disrupt their own circadian rhythm after the onset of the disease, and the disorganization of the circadian rhythm will result in a poor prognosis of the patients [80]. Furthermore, upregulation of *KLF15* expression after myocardial infarction has been shown to improve the damage [61]. The perspective of *KLF15* and circadian rhythm provides new research direction and therapeutic ideas for the treatment of myocardial infarction [Figure 1].

Upon receiving light signals, the SCN modulates *KLF15*-regulated cardiac rhythms through both autonomic neural pathways and hormonal signaling pathways. *KLF15* orchestrates energy metabolism during the active phase and facilitates damage repair during the resting phase. The morning peak incidence of myocardial infarction results from coordinated effects of arrhythmias, morning blood pressure surge, platelet activation, and inhibited fibrinolysis—all jointly regulated by core clock genes and *KLF15*.

## 5. Limitations and Future Research Directions

Since this review primarily utilizes rodent models to elucidate the role of *KLF15*-mediated circadian rhythms in myocardial infarction (MI), while rodent models have provided valuable mechanistic insights—such as *KLF15*’s regulation of cardiac metabolism and inflammation—their applicability to human pathophysiology is constrained by critical differences. Therefore, it is essential to acknowledge significant translational gaps. From the perspective of circadian physiology, humans exhibit unique circadian behaviors (e.g., sleep-wake cycles, hormonal fluctuations) and environmental exposures (e.g., artificial light, shift work), which cannot be fully replicated in rodents. For instance, the morning peak in MI incidence in humans may involve complex interactions between neural activation and systemic stress factors (e.g., abrupt blood pressure surges), whereas these pathways have not been closely examined in rodent models. Additionally, although *KLF15* is conserved across species, its rhythmic expression and downstream targets in human cardiomyocytes remain poorly characterized. Human-based studies are scarce, and existing data mainly rely on postmortem tissues or in vitro models, which may fail to capture dynamic circadian interactions. Furthermore, rodent models typically focus on a single disease condition, neglecting systemic states, which differs from humans who often present with comorbidities. Metabolic disorders, in particular, may disrupt *KLF15*’s circadian regulation, thereby altering its cardioprotective effects.

To enhance clinical relevance, future studies should incorporate time-dependent sampling of patient blood or tissues to correlate *KLF15* expression rhythms with MI timing and patient outcomes. Furthermore, human induced pluripotent stem cell (iPSC)-derived cardiomyocyte models could be integrated with microfluidic chip technology to better mimic human physiological conditions. Given that existing *KLF15*-targeting drugs may have off-target effects and gene therapy remains underdeveloped, non-pharmacological interventions such as acupuncture warrant exploration. Emerging evidence suggests that acupuncture can modulate biological clock genes and enhance drug efficacy [81], with a favorable safety profile. If rigorously validated in clinical trials, acupuncture may emerge as a breakthrough adjuvant therapy for synchronizing *KLF15* rhythms in MI secondary prevention. *KLF15*, as a Therapeutic Target for Circadian-Based Cardiac Repair and a circadian checkpoint in the heart, *KLF15* maintains rhythmic gene expression critical for cardiac health, influencing MI progression and recovery. Therefore, *KLF15* represents a promising therapeutic target, with potential strategies including:

(1) Restoring *KLF15* Oscillation: Reprogramming downstream gene networks by reinstating *KLF15*’s circadian rhythm to facilitate myocardial repair.
(2) Harnessing *KLF15*’s Multifunctional Effects: Leveraging its anti-inflammatory, antioxidant, and metabolic regulatory properties to mitigate inflammation, reduce cardiomyocyte death, limit infarct expansion, and prevent further cardiac dysfunction.
(3) Timed Intervention Strategies: Prolonging *KLF15* expression during its peak or trough phases to induce compensatory upregulation and prevent infarction.

These chronotherapeutic approaches not only deepen our understanding of cardiac disease mechanisms but also provide a theoretical and practical framework for time-precise treatments. Further validation in murine models is essential to advance clinical translation, offering novel strategies to address critical public health challenges in cardiovascular disease.

## 6. Conclusions

**Figure 2:**
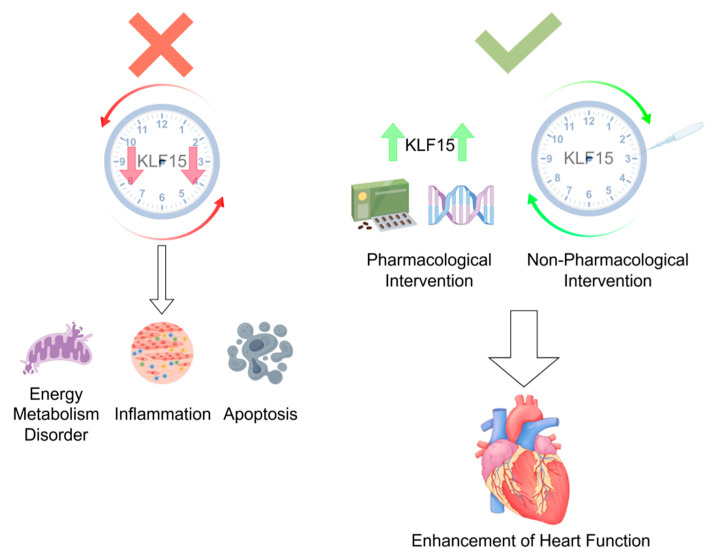
*KLF15*’s regulatory role in the heart. By Figdraw.

The pivotal role of *KLF15* in mediating circadian regulation of myocardial infarction (MI) underscores the profound influence of biological rhythms on cardiovascular health and disease. *KLF15* dysfunction disrupts cardiac metabolic homeostasis, exacerbates inflammatory injury, and impairs post-infarction repair, while its rhythmic restoration offers significant therapeutic promise. As mechanistic insights into *KLF15*’s temporal regulation deepen, this transcription factor may emerge as a key target for MI prevention and treatment. Future research should employ advanced translational approaches to bridge the gap between preclinical findings and clinical applications. Multivariate analyses could determine whether *KLF15* expression patterns or genetic variants serve as prognostic biomarkers for MI susceptibility or recovery. Moreover, therapeutic strategies should capitalize on *KLF15*’s circadian properties, such as timed pharmacological interventions or lifestyle modifications that synchronize its rhythmic activity. By integrating chronobiological principles with cardiovascular medicine, *KLF15*-centered therapies may revolutionize MI management—transforming circadian biology from a conceptual framework into actionable clinical strategies. These innovations hold the potential to improve patient outcomes and pave the way for personalized, time-sensitive cardiovascular care [Figure 2].

When KLF15 rhythm is disturbed or its expression is reduced, it will cause more serious abnormalities of energy metabolism, inflammation and apoptosis. Up-regulation of KLF15 concentration by pharmacologic or non-pharmacologic therapies corrects KLF15 rhythm disturbances and improves cardiac function after myocardial infarction.

## Figures and Tables

**Table 1:** Key References in the Review. MI (myocardial infarction); NF-κB (Nuclear Factor kappa-light-chain-enhancer of activated B cells); MAPK (Mitogen-Activated Protein Kinase); PI3K-AKT (Phosphoinositide 3-Kinase-Protein Kinase B Signaling Pathway); PPARγ (Peroxisome Proliferator-Activated Receptor Gamma); BCAA (Branched-chain amino acids); BCKA (Branched-chain keto acids).

Reference	Year	Author	Key Findings
[1]	2024	Liu, M.; He, X.; Wang, Z.; Hu, S.	Provides data on the epidemiology of cardiovascular diseases and the current status of interventional diagnosis and treatment in China.
[3]	2007	Thygesen, K.; Alpert, J. S.; White, H. D.; Jaffe, A. S.; Apple, F. S.; Galvani, M.; Katus, H. A.; Newby, L. K.; Ravkilde, J.; Chaitman, B.; et al.	International diagnostic criteria for myocardial infarction.
[5]	2022	Sagris, M.; Antonopoulos, A. S.; Theofilis, P.; Oikonomou, E.; Siasos, G.; Tsalamandris, S.; Antoniades, C.; Brilakis, E. S.; Kaski, J. C.; Tousoulis, D.	Adverse lifestyle behaviors, including chronic sleep deprivation, markedly elevate the incidence of acute myocardial infarction among young adults.
[6]	2023	Rabinovich-Nikitin, I.; Kirshenbaum, E.; Kirshenbaum, L. A.	Reveals the critical role of the biological clock-autophagy axis in cardiovascular diseases, providing a theoretical foundation for chronotherapy.
[7]	2025	Delisle, B. P.; Prabhat, A.; Burgess, D. E.; Stumpf, I. G.; McCarthy, J. J.; Procopio, S. B.; Zhang, X.; Esser, K. A.; Schroder, E. A.	elucidates the molecular mechanism by which the biological clock regulates ion channels via *KLF15*, thereby explaining the morning peak incidence of myocardial infarction and cardiac arrhythmias.
[10]	2011	Wu, X.; Liu, Z.; Shi, G.; Xing, L.; Wang, X.; Gu, X.; Qu, Z.; Dong, Z.; Xiong, J.; Gao, X.; et al.	This study provides direct evidence in animal models that biological clock genes dynamically regulate cardiac function in real time.
[19]	2015	Zhang, L.; Prosdocimo, D. A.; Bai, X.; Fu, C.; Zhang, R.; Campbell, F.; Liao, X.; Coller, J.; Jain, M. K.	*KLF15* orchestrates cardiac metabolism and repair through a biphasic regulatory mode, thereby providing mechanistic evidence for the circadian nature of myocardial infarction.
[21]	1996	Molnar, J.; Zhang, F.; Weiss, J.; Ehlert, F. A.; Rosenthal, J. E.	This study establishes a clinically relevant link between QTc rhythm variations and temporal patterns of cardiovascular events, providing evidence for the dual regulation of cardiac electrophysiology by both the autonomic nervous system and biological clock.
[22]	2007	Culić, V.	Myocardial infarction exhibits circadian rhythmicity, correlating with neural activation and blood pressure fluctuations.
[23]	2016	Takeda, N.; Maemura, K.	The *Bmal1*/*CLOCK* genes regulate circadian blood pressure rhythms, and their deficiency causes rhythm disruption, thereby increasing myocardial infarction risk.
[24]	1989	Kurihara, H.; Yamaoki, K.; Nagai, R.; Yoshizumi, M.; Takaku, F.; Satoh, H.; Inui, J.; Yazaki, Y.	Endothelin-1 levels exhibit a morning surge, which may contribute to the higher morning incidence of myocardial infarction.
[25]	2006	Förstermann, U.; Münzel, T.	Oxidative stress reduces endothelial nitric oxide synthase (eNOS) activity, leading to increased vascular tone in the morning and consequently exacerbating cardiovascular risk.
[26]	2014	Scheer, F. A.; Shea, S. A.	The morning peak of plasminogen activator inhibitor-1 (PAI-1) increases thrombotic risk and may underlie the morning predominance of myocardial infarction.
[27]	2011	Scheer, F. A.; Michelson, A. D.; Frelinger, A. L., 3rd; Evoniuk, H.; Kelly, E. E.; McCarthy, M.; Doamekpor, L. A.; Barnard, M. R.; Shea, S. A.	The surface activity of platelet GPIIb-IIIa complex peaks during circadian morning hours, enhancing thrombus formation and potentially explaining the morning peak incidence of myocardial infarction.
[28]	1995	Knutsson, A.	Circadian rhythm disruption elevates myocardial infarction risk.
[29]	1995	Kirshenbaum, L. A.; Hill, M.; Singal, P. K.	Circadian disruption exacerbates oxidative stress, rendering cardiomyocytes more vulnerable to injury.
[30]	2011	Mann, D. L.	Circadian regulation of immune responses affects post-MI repair.
[31]	2021	Rabinovich-Nikitin, I.; Rasouli, M.; Reitz, C. J.; Posen, I.; Margulets, V.; Dhingra, R.; Khatua, T. N.; Thliveris, J. A.; Martino, T. A.; Kirshenbaum, L. A.	The *Bmal1* gene enhances mitophagy to protect ischemic cardiomyocytes and improve survival rates.
[32]	2014	Alibhai, F. J.; Tsimakouridze, E. V.; Chinnappareddy, N.; Wright, D. C.; Billia, F.; O’Sullivan, M. L.; Pyle, W. G.; Sole, M. J.; Martino, T. A.	Light/dark cycle disruption exacerbates post-MI cardiac remodeling and impairs functional recovery in mice.
[33]	2014	Kohsaka, A.; Das, P.; Hashimoto, I.; Nakao, T.; Deguchi, Y.; Gouraud, S. S.; Waki, H.; Muragaki, Y.; Maeda, M.	*Bmal1* deficiency dysregulates cardiac oxidative stress-related gene expression and exacerbates myocardial infarction injury.
[34]	2020	Li, L.; Li, H.; Tien, C. L.; Jain, M. K.; Zhang, L.	*KLF15* maintains normal ventricular rhythm by regulating the circadian oscillation of cardiac ion channels and QT interval.
[35]	2012	Jeyaraj, D.; Scheer, F. A.; Ripperger, J. A.; Haldar, S. M.; Lu, Y.; Prosdocimo, D. A.; Eapen, S. J.; Eapen, B. L.; Cui, Y.; Mahabeleshwar, G. H.; et al.	*KLF15* serves as a pivotal link between circadian rhythms and cardiac function.
[36]	2021	He, S.; Lu, Y.; Guo, Y.; Li, S.; Lu, X.; Shao, S.; Zhou, H.; Wang, R.; Wang, J.; Gao, P.; et al.	*KLF15* exerts anti-inflammatory effects via NF-κB/MAPK inhibition.
[37]	2021	Wang, B.; Xu, H.; Kong, J.; Liu, D.; Qin, W.; Bai, W.	*KLF15* attenuates ischemic apoptosis via p38/MAPK inhibition.
[38]	2019	Zhou, L.; Li, Q.; Chen, A.; Liu, N.; Chen, N.; Chen, X.; Zhu, L.; Xia, B.; Gong, Y.; Chen, X.	*KLF15*-Twist2 axis inhibits inflammation and enhances mitochondrial homeostasis.
[39]	2022	Liao, B.; Tian, X.	*KLF15* alleviates ischemia-reperfusion injury by modulating CTRP12 expression.
[40]	2024	Zheng, H.; Ye, W.; Huang, K.; Chen, Q.; Yang, J.; Luo, L.	KLF15 activates PI3K-AKT to enhance cytoprotection against oxidative damage.
[41]	2014	Prosdocimo, D. A.; Anand, P.; Liao, X.; Zhu, H.; Shelkay, S.; Artero-Calderon, P.; Zhang, L.; Kirsh, J.; Moore, D.; Rosca, M. G.; et al.	*KLF15*-p300 axis attenuates cardiac lipotoxicity by reprogramming lipid metabolism.
[42]	2011	Nagare, T.; Sakaue, H.; Matsumoto, M.; Cao, Y.; Inagaki, K.; Sakai, M.; Takashima, Y.; Nakamura, K.; Mori, T.; Okada, Y.; et al.	*KLF15* optimizes cardiac energetics via insulin-GLUT4 axis.
[43]	2024	Durumutla, H. B.; Prabakaran, A. D.; El Abdellaoui Soussi, F.; Akinborewa, O.; Latimer, H.; McFarland, K.; Piczer, K.; Werbrich, C.; Jain, M. K.; Haldar, S. M.; et al.	Glucocorticoid-*KLF15* circadian axis coordinates cardiac glucose utilization.
[44]	2015	Jiao, J.; Zou, S.; Jian, C.; Tang, F.; Xiao, Y.; Chen, L.	*KLF15* enhances post-unloading cardiac recovery by suppressing lipid accumulation.
[45]	2022	Hu, Y.; Xu, J.; Gao, R.; Xu, Y.; Huangfu, B.; Asakiya, C.; Huang, X.; Zhang, F.; Huang, K.; He, X.; et al.	*KLF15* attenuates lipid accumulation via PPARγ downregulation.
[46]	2016	Sun, H.; Olson, K. C.; Gao, C.; Prosdocimo, D. A.; Zhou, M.; Wang, Z.; Jeyaraj, D.; Youn, J. Y.; Ren, S.; Liu, Y.; et al.	*KLF15* deficiency disrupts BCAA catabolism, causing BCKA-induced mitochondrial impairment in heart failure.
[47]	2023	Zhu, X.; Liu, J.; Cai, M.; Bao, L.; Pan, Y.; Wu, P.; Chu, W.; Zhang, J.	Fasting-induced *Clock*-*KLF15*-Bcat2 axis dysregulation impairs BCAA metabolism in fish muscle.

**Table 2:** The multiple roles of *KLF15*.

	Key Point	Reference(s)	Observed Effect on MI
*KLF15*-Inflammations	NF-κB	He, S et al. (2021) [36]	*KLF15* is able to attenuate inflammatory responses by inhibiting NF-κB pathway activation
		Huang, B et al. (2010) [58]	
	MAPK	Wang, B et al. (2021) [37]	*KLF15* is able to attenuate inflammatory responses by inhibiting MAPK pathway activation
	Twist2	Zhou, L et al. (2019) [38]	*KLF15* ameliorates postmyocardial infarction injury by regulating Twist2
	CTRP12	Liao, B et al. (2022) [39]	*KLF15* can alleviate post-ischemic reperfusion injury by regulating CTRP1250
*KLF15*-Apoptosis	PI3K-AKT	Zheng, H et al. (2024) [40]	*KLF15* ameliorates apoptosis through the PI3K-AKT pathway
*KLF15*-EnergyMetabolism	Glucose Metabolism	Nagare, T et al. (2011) [42]	*KLF15* promotes the entry of glucose into cardiomyocytes for cardiac energy supply by regulating insulin secretion
	Lipid Metabolism	Fan, L et al. (2022) [72]	*KLF15* maintains lipid homeostasis in the heart by regulating PPAR-δ and PPAR-γ
		Gire, D et al. (2021) [73]	
		Hu, Y et al. (2022) [45]	
	Branched-Chain AminoAcid Metabolism	Sun, H et al. (2016) [46]	*KLF15* improves cardiac function by regulating BCKAs
		Jeyaraj, D et al. (2012) [35]	

## Data Availability

This review article is based on previously published studies. All data analyzed in this work are publicly available through the cited references. No new datasets were generated or analyzed during this review. Links to the original sources are provided in the reference list.
